# Point-of-care lung ultrasound imaging in pediatric COVID-19

**DOI:** 10.1186/s13089-020-00198-z

**Published:** 2020-11-30

**Authors:** Eliana P. C. Giorno, Milena De Paulis, Yoshino T. Sameshima, Kirstin Weerdenburg, Paulo Savoia, Danilo Y. Nanbu, Thomaz B. Couto, Fernanda V. M. Sa, Sylvia C. L. Farhat, Werther B. Carvalho, Marcela Preto-Zamperlini, Claudio Schvartsman

**Affiliations:** 1 Emergency Department, Instituto da Criança da FMUSP, University of Sao Paulo, Av. Dr. Enéas Carvalho de Aguiar, 647, Cerqueira César, Sao Paulo, SP CEP-05403.000 Brazil; 2 Emergency Department, Hospital Universitario, University of Sao Paulo, Sao Paulo, Brazil; 3 Emergency Department, Hospital Israelita Albert Einstein, Sao Paulo, Brazil; 4 Radiology Department, Hospital Israelita Albert Einstein, Sao Paulo, Brazil; 5 Dalhousie University, Halifax, NS Canada; 6 IWK Health Centre, Halifax, NS Canada; 7 Radiology Department, Radiology Institute, University of Sao Paulo, Sao Paulo, Brazil; 8 Pediatric Intensive Care, Children’s Institute, University of Sao Paulo, Sao Paulo, Brazil

**Keywords:** COVID-19, Lung ultrasound, Point-of-care, Children

## Abstract

**Background:**

There has been limited data regarding the usefulness of lung ultrasound (US) in children with COVID-19.

**Objective:**

To describe lung US imaging findings and aeration score of 34 children with COVID-19.

**Methods:**

This study included 0–16-year-old patients with confirmed COVID-19, who were admitted between April 19 and June 18, 2020 in two hospitals in the city of Sao Paulo, Brazil. Lung US was performed as part of the routine evaluation by a skilled Pediatric Emergency physician. Clinical and laboratory data were collected and severity classifications were done according to an available clinical definition. The lung US findings were described for each lung field and a validated ultrasound lung aeration score was calculated. Data obtained was correlated with clinical information and other imaging modalities available for each case.

**Results:**

Thirty-four confirmed COVID-19 patients had a lung US performed during this period. Eighteen (18/34) had abnormalities on the lung US, but eight of them (8/18) had a normal chest radiograph. Ultrasound lung aeration score medians for severe/critical, moderate, and mild disease were 17.5 (2–30), 4 (range 0–14), 0 (range 0–15), respectively (*p* = 0.001). Twelve patients (12/34) also had a chest computed tomography (CT) performed; both the findings and topography of lung compromise on the CT were consistent with the information obtained by lung US.

**Conclusion:**

Point-of-care lung US may have a key role in assessing lung injury in children with COVID-19.

## Introduction

Since its initial identification in December 2019, the coronavirus disease 2019 (COVID-19) has infected millions of people and has led to thousands of deaths worldwide as of July 2020 [1]. Although reports from adults suggest that this disease can have a more severe course in as many as 18.5% of infected people, in children it seems to be milder with an estimated incidence of severe cases of 4–6% and a lower case-fatality ratio reported [1–3]. Previous studies regarding the clinical aspects of COVID-19 in children described symptoms that followed a similar pattern as in adults [3], but recent reports have identified an emerging novel spectrum of the disease in children, which includes a multisystem inflammatory condition with overlapping features of toxic shock syndrome [4]. Up to now, the absolute number of those cases with prominent cardiovascular compromise is still low and respiratory symptoms remains the main reason for Pediatric Intensive Care Unit (PICU) admissions for COVID-19 [5].

Efforts have been made to create guidelines for the diagnosis, treatment, and prevention of COVID-19 in children [6, 7]. Of particular concern is which imaging modality is most suitable to assess the extension of lung involvement. Historically, chest radiograph has been the imaging modality of choice for most lower respiratory illnesses in children, but data from adult studies have shown that for COVID-19 it is of limited value and is not recommended as the first choice for imaging modalities [8]. Often, only the severe cases show abnormalities on chest radiographs [6]. In earlier stages of the infection, it frequently fails to show the typical features of COVID-19 that are found with chest computed tomography (CT) [9, 10].

Chest CT is undoubtedly the best imaging modality to accurately assess lung involvement in most respiratory illnesses including COVID-19, but the cost and possible harmful effects that radiation can have on a growing child’s body must not be ignored, as even low-dose ionizing radiations may increase cancer risk in exposed children [11].

Lung ultrasound (US) is an ionizing radiation-free imaging modality that promptly provides bedside diagnosis of many pulmonary conditions in the emergency department. It has been reported to be highly accurate and reliable in diagnosing pneumonia, pleural effusion, and pneumothorax [12–14]. In patients who are mechanically ventilated, lung US enables clinicians to monitor lung aeration and its variations. It has been found that different US patterns correspond to different degrees of aeration loss, which led to the creation of a score that was subsequently validated to quantify lung aeration as a whole or in a given area of interest [15]. Compared to chest CT as the gold standard, the aforementioned lung US score has been useful in monitoring aeration in the settings of Acute Respiratory Distress Syndrome (ARDS) and Ventilator-Associated Pneumonia [16, 17].

Given the already widely acknowledged usefulness of lung US to detect several lung pathologies in acutely ill patients and its ability to estimate lung aeration, we decided to incorporate this imaging modality into our Pediatric Emergency Department (PED) observation unit as part of the routine evaluation of children admitted with confirmed COVID-19. In this study, we describe the lung US findings in these patients with COVID-19 and correlate it with clinical severity.

## Methods

### Design, setting and participants

This study included 0–16-year-old patients with laboratory-confirmed COVID-19 infection, who were admitted to two academic hospitals in the city of Sao Paulo, Brazil, between April 19 and June 18, 2020. The participating hospitals included one secondary care center and one tertiary care center, both from the University of Sao Paulo. The latter participating hospital has become a reference for the management of moderate and severe cases of COVID-19 in the state of Sao Paulo.

A confirmed case of COVID-19 was defined by a positive result on the RT-PCR assay of a specimen collected from a nasopharyngeal swab or a positive IgG and IgM antibodies specific for SARS-Cov-2, titled using the viral nucleoprotein as the antigen in an ELISA test in-house.

Point-of-care lung US is part of the routine evaluation carried out for children with respiratory illnesses in the PED of these institutions and is present on the evaluation flowchart of COVID-19 children as an option for an imaging assessment. When a patient had confirmed COVID-19, lung US was performed regardless of the symptoms reported. Patients with chronic lung disease or lung metastasis who had undergone a lung US were not included in this study. Patients with other chronic diseases or cancer without lung metastasis were not excluded.

We collected data on age, sex, clinical symptoms, and signs at presentation, coexisting or chronic conditions, laboratory and radiologic results, and the support needed during hospital admission. Patients were classified as mild, moderate, severe, or critical according to a clinical definition suggested by Qiu et al. [18] Laboratory and other imaging tests that were also performed, including chest radiograph and chest CT were requested based on each institution’s guidelines and at the discretion of the treating physician.

*Point-of-care lung ultrasound.* It was performed by one of the five trained Pediatric Emergency (PEM) Physicians with more than 100 lung scans performed, using B-mode imaging, basic preset, adjusting depth, and 2D-gain according to each patient biotype. A high-frequency linear transducer (15 MHz) was used and the technique was similar to that described by Copetti and Catarossi [19] in which all intercostal spaces of the upper and lower parts of the anterior, lateral, and posterior regions of the left and right chest are examined, making a total of 12 regions. To describe the findings and to calculate the lung US aeration score, the worst US finding was considered in each examined region. To evaluate interobserver reproducibility, images of 25 patients enrolled were reviewed and scored by a different PEM of the team, who was blinded to the clinical information.

Four validated US patterns were defined and a score given for each: [16, 17] (1) normal aeration: presence of lung sliding and artifactual horizontal A-lines (0 points); (2) loss of lung aeration resulting from the scattered foci of bronchopneumonia or interstitial syndrome: presence of multiple well-defined vertical B-lines extending from the pleural line or a small subpleural consolidation (1 point); (3) loss of lung aeration resulting from alveolar-interstitial edema that corresponds to the CT imaging entity of the ground-glass: multiple confluent vertical B-lines extending from the pleural line or a small subpleural consolidation (2 points); (4) lung consolidation characterizing extensive bronchopneumonia: presence of a tissue structure containing hyperechoic punctiform images representative of air bronchograms (3 points). A global lung US aeration score ranging from 0 to 36 was obtained by summing the individual scores of all the regions.

All medical investigation procedures described were conducted as part of the standard clinical care. This research was part of a longitudinal study approved by the local ethics board.

### Statistical analysis

The baseline patient characteristics were expressed as absolute and relative frequencies for qualitative variables and as median, minimum, maximum, mean and standard deviation for quantitative variables. The non-parametric Kruskal–Wallis *H*-test was applied to compare the lung US scores in relation to severity classification. In addition, we performed a post hoc analysis using the Bonferroni multiple comparison procedure. Interobserver agreements in lung aeration score determined for each region of interest were assessed using the kappa coefficient test. The significance level was fixed at 5% for all the tests. Statistical analyses were performed using IBM SPSS Statistics version 23.0 (IBM Corp., Armonk, NY, USA) and R software version 3.5 (R Foundation for Statistical Computing, Vienna, Austria).

## Results

### Clinical and laboratory characteristics of the patients

From April 19 to June 18, 2020, 34 admitted patients with confirmed COVID-19 had a lung US performed and were included in the study. During this period, a total of 51 pediatric patients with COVID-19 diagnosis were admitted. Lung US was performed depending on the availability of a skilled PEM physician in the PED observation unit or PICU. Of these 34 patients that were scanned, 33 had a positive RT-PCR assay and one had positive serology in a context of clinical suspicion. A summary of the clinical features is detailed in Table 1. The mean duration of symptoms in days before lung US was performed was 3.74 ± 1.76 and median 3 (range 1–8). The most common symptom was fever, followed by cough and shortness of breath. Five patients had critical disease (15%) and required respiratory and hemodynamic support. Three were classified as severe (9%), eight (23%) as moderate, and the remaining presented with mild disease (53%) but were admitted due to underlying chronic conditions or their young age.

**Table 1:** Summary of the clinical features of the patients

Variables studied	Clinical classification			
	Mild (*n*:18)	Moderate (*n*:8)	Severe/critical (*n*:8)	Total (*n*:34)
Epidemiological data				
Age, months Median (range)	23 (0.46–192)	4 (1–132)	29 (0.3–132.16)	13 (0,3–192)
Male sex *n* (%)	14 (78)	4 (50)	3 (37)	21 (62)
Chronic medical condition *n* (%)	9 (50)	6 (75)	3 (37)	18 (53)
Symptoms *n* (%)				
Fever	18 (100)	7 (87)	7 (87)	32 (94)
Rhinorrhea	5 (27)	4 (50)	0	9 (26)
Cough	5 (27)	5 (62)	7 (87)	17 (50)
Dyspnea	0	8 (100)	7 (87)	15 (44)
Nausea/vomiting	2 (11)	0	2 (25)	4 (12)
Respiratory failure	0	0	7 (87)	7 (20)
Shock	0	0	5 (62)	5 (15)
Hypoxemia	0	0	8 (100)	8 (23)
Early cardiac failure^a^	0	0	2 (25)	2 (6)
Pulmonary edema	0	0	1(12)	1 (3)
Laboratorial data				
D-Dimer (ng/mL) Median (range)	1251 (306–5809)	1990 (190–4127)	18,536 (1932–54,153)	2250 (190–54,153)
C-Reactive Protein (mg/L) Median (range)	5.4 (0.3–130)	3 (0.5–114)	185.5 (0.3–447)	20 (0.3–447)
Intensive care support *n* (%)				
Mechanical ventilation	0	0	5 (62)	5 (15)
Vasoactive drug	0	0	5 (62)	5 (15)
Imaging data *n* (%)				
Chest RX performed	12(66)	6 (75)	7 (87)	25 (73)
Chest RX abnormality	0	1/6 (16)	6/7(85)	7/25 (28)
Chest CT performed	3 (16)	3 (37)	6 (75)	12 (35)
Chest CT abnormality	1/3 (33)	3/3 (100)	6/6 (100)	10/12 (83)
Lung US score^b^ Median (range)	0 (0–15)	4 (0–14)	17 (2–30)	2,5 (0–30)

^a^ Present early after hospital admission, at the time lung US was performed

^b^ *p* value < 0.001

The critical/severe group was composed of eight children, all of them with prominent respiratory disease. Seven out of these eight patients had echocardiography performed by a cardiologist or a focused cardiac exam performed by an intensive care physician. Systolic dysfunction was initially found in only two patients who also had consolidations on lung US and CT. The remaining five had a normal biventricular systolic function at the time of lung US, but three of them later developed shock probably due to worsening disease and elevated pressures on mechanical ventilation. Three critical patients have died. One due to cardiac failure and two due to ARDS.

Baseline medical conditions were common in our population and were present in 18 patients (53%); eight of them with cancer (8/18—44%), the remaining patients had chronic liver, kidney or neurologic/genetic disease. D-Dimer, a severity marker, was collected in 20/34 (59%) patients with a median of 2250.5 (190–54,153); 17 of them (17/20—85%) had elevated values, although symptoms were mild in seven out of these 17 (41%).

### Imaging findings

Lung US abnormalities were found in 18 (53%) patients and these findings are described in Table 2. Of the 18 patients with lung US abnormalities, eight had severe/critical disease (44%), five had moderate disease (28%) and five (28%) had mild disease. All the patients with lung US abnormalities but who had a mild disease were previously healthy infants under 6 months of age (mean 1.24 ± 0.89), with fever and mild respiratory symptoms. Three of them were also tested for other 17 viruses, with negative results. Their median lung US aeration score was 12 (3–15).

**Table 2:** Summary of lung the US findings, lung US aeration score, and CT findings

Patient	Severity	Lung US Day^a^	Lung US main findings	Lung US score	CT Day^a^	CT finding
1	Mild	Day 3	Multiple well-defined B-lines in all the regions bilaterally; Confluent B-lines in the anterior and lateral bases and posterior upper right lobe; confluent B-lines on the lateral left base with pleural disruption	15		–
2	Mild	Day 3	Normal	0		–
3	Critical	Day 5	Consolidations > 0.5 cm in the anterior, lateral and posterior right lung, superior and inferior; Multiple confluent B-lines in the anterior base, lateral and posterior left lung with consolidation .0,5 cm in the posterior left base; Bilateral pleural effusion	30	Day 15	Foci of consolidation sometimes rounded, associated with multifocal ground-glass opacities seen bilaterally predominantly of peripheral distribution Small pleural effusion
4	Moderate	Day 5	Posterior right upper lobe subpleural consolidation < 0.5 cm with confluent B-lines	4	Day 4	Ground-glass opacity on the posterior right upper lobe
5	Mild	Day 3	Normal	0		–
6	Moderate	Day 2	Normal	0		–
7	Mild	Day 4	Normal	0		–
8	Mild	Day 4	Multiple well-defined B-lines bilaterally. Confluent B-lines in posterior left lung with pleural disruption and subpleural consolidations < 0.5 cm	12	Day 6	Multiple lung opacities and scarce foci of consolidation bilaterally, predominantly of peripheral distribution and in the left lower lobe
9	Critical	Day 7	Confluent B-lines on the lateral lung field bilaterally Consolidations > 0.5 cm of the posterior lung field bilaterally Bilateral pleural effusion	20	Day 7	Multiples ground-glass opacities, with interlobular septum thickening and multiple areas of consolidation, predominantly of peripheral distribution and in the posterior and inferior lung fields bilaterally Bilateral pleural effusion
10	Mild	Day 2	Normal	0		–
11	Mild	Day 2	Normal	0		–
12	Mild	Day 2	Normal	0	Day 3	Normal
13	Critical	Day 6	Scarce multiple well-defined B-lines in the anterior and lateral right lung; confluent B-lines and subpleural consolidation in the posterior right lung. Confluent B-lines in the posterior left lung	12	Day 7	Consolidation and centrilobular opacities with ground-glass attenuation in the upper and lower lobe of the right lung. Parenquimatous band in the left lower lobe
14	Moderate	Day 2	Confluent B-lines in the posterior bases bilaterally, with < 0.5 cm subpleural consolidation	4		–
15	Mild	Day 4	Multiple well-defined B-lines in the right posterior upper lobe; Confluent B-lines in the posterior right lower lobe	3		–
16	Mild	Day 4	Multiple well-defined B-lines on the right lung and anterior left lung; Confluent B-lines on the lateral left base and posterior left lung	15		–
17	Mild	Day 3	Normal	0		–
18	Mild	Day 3	Multiple well-defined B-lines on the anterior bases bilaterally; Consolidation > 0.5 cm in the left upper posterior lung	5		–
19	Moderate	Day 8	Multiple well-defined B-lines in the right lung Confluent B-lines and consolidation > 0.5 cm in the posterior left base	8	Day 14	Parenquimatous band in the upper lobes and inferior left lobe, with a small consolidation on the left base
20	Mild	Day 4	Normal	0	Day 5	Normal
21	Mild	Day 1	Normal	0		–
22	Moderate	Day 2	Confluent B-lines and < 0.5 cm subpleural consolidations in the right posterior upper and lower lung. Multiple well-defined B-lines and pleural disruption on the left lung, confluent B-lines on left lung base	14	Day 3	Ground-glass opacities with consolidation and atelectasis bilaterally, more prominent in the right posterior upper and lower lung
23	Mild	Day 2	Normal	0		–
24	Mild	Day 7	Normal	0		–
25	Critical	Day 3	Multiple well-defined B-lines in the lateral and posterior right lung; confluent B-lines on anterior upper right lung; Multiple well-defined B-lines on the left lung with consolidations > 0.5 cm in the lateral and posterior upper left lobe	15		–
26	Moderate	Day 3	Multiple well-defined B-lines in all the lung fields bilaterally	12		–
27	Mild	Day 2	Normal	0		–
28	Moderate	Day 5	Normal	0		
29	Severe	Day 7	Multiple well-defined B-lines in the anterior lung fields bilaterally. Confluent B-lines on posterior lung fields bilaterally and lateral right base; < 0.5 cm subpleural consolidations in the posterior right lung	14	Day 9	Ground-glass opacities bilaterally; few small consolidations on the right lung, upper and inferior lobes
30	Severe	Day 5	Confluent B-lines in the posterior upper right lobe	2		
31	Mild	Day 4	Normal	0		
32	Moderate	Day 3	Normal	0		
33	Critical	Day 2	Multifocal confluent B-lines and consolidations > 0.5 cm on posterior and lateral lung fields bilaterally	20	Day 3	Multiple ground-glass opacities and scattered foci of consolidations, predominantly of peripheral distribution in the posterior lung fields bilaterally
34	Severe	Day 5	Confluent B-lines and < 0.5 cm consolidations on anterior lateral and posterior lung fields bilaterally	21	Day 6	Multiples ground-glass opacities, with interlobular septum thickening and multiple areas of consolidation, predominantly of peripheral distribution

^a^Time elapsed, since the symptoms began

Seventeen (17/18—94%) patients had confluent vertical B-lines on at least one posterior region; this pattern was found bilaterally in the posterior lung fields in 9/18 (50%) patients. Six out of 18 (33%) patients also had lung consolidations > 0.5 cm. Each patient’s lung US aeration scores are depicted in Table 2. Mean lung US scores for severe/critical, moderate and mild cases were 16.75 ± 8.16, 5.25 ± 5.55 and 2.78 ± 5.36, respectively. When multiple comparisons were made, only mild versus severe/critical met statistical significance (*p* < 0.0001). Interobserver agreement was good, as attested by kappa coefficient of 0.71 (*p* < 0.001).

Chest radiograph was obtained in 25/34 patients (73%); only six with severe/critical disease and one with moderate disease showed pulmonary opacities; the remaining 18 were declared to be normal by the treating physician. Eight patients with lung abnormalities on lung US had a normal chest radiograph, but no patients with normal lung US had abnormalities on the chest radiograph.

Chest CT was performed in 12 patients (12/34—35%). Ten had abnormalities and two were normal. The most common feature was bilateral ground-glass opacities. After being carefully reviewed by two experienced radiologists, both findings and topography of the abnormalities on lung US were found to be similar to those of CT. More details on the CT findings and the corresponding lung US findings are described in Table 2. The time that elapsed from lung US and chest CT was less than 24 h for eight patients and more than 24 h for four patients; one of these four patients was unstable and could not be transported to the radiology suite earlier.

**Fig. 1:**
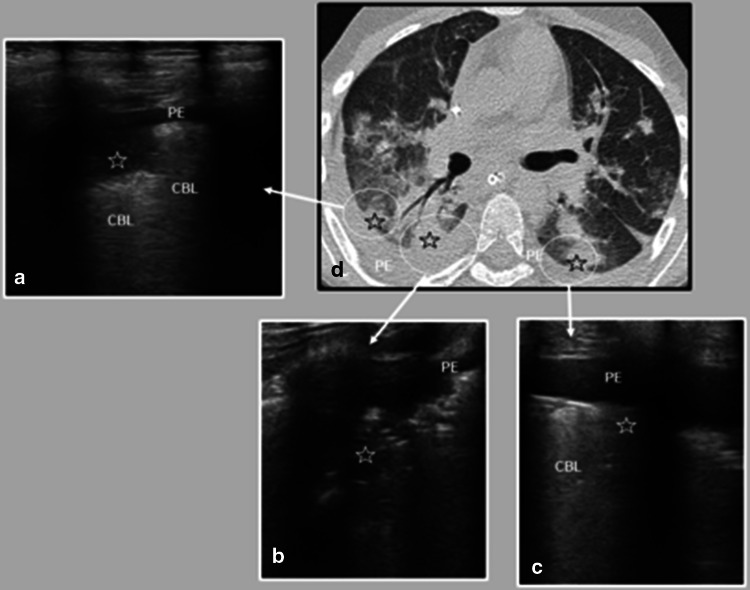
Eleven-year-old male with respiratory failure and shock. Lung US (**a**–**c**) was performed on the 5th day after symptom onset and chest CT (**d**) on the 15th day. There was a pleural effusion (PE in **a**–**d**) and subpleural consolidations (stars in **a**–**d**). Confluent B-lines (CBL in **a** and **c**) were also present in the lung US, corresponding to the chest CT ground-glass pattern

**Fig. 2:**
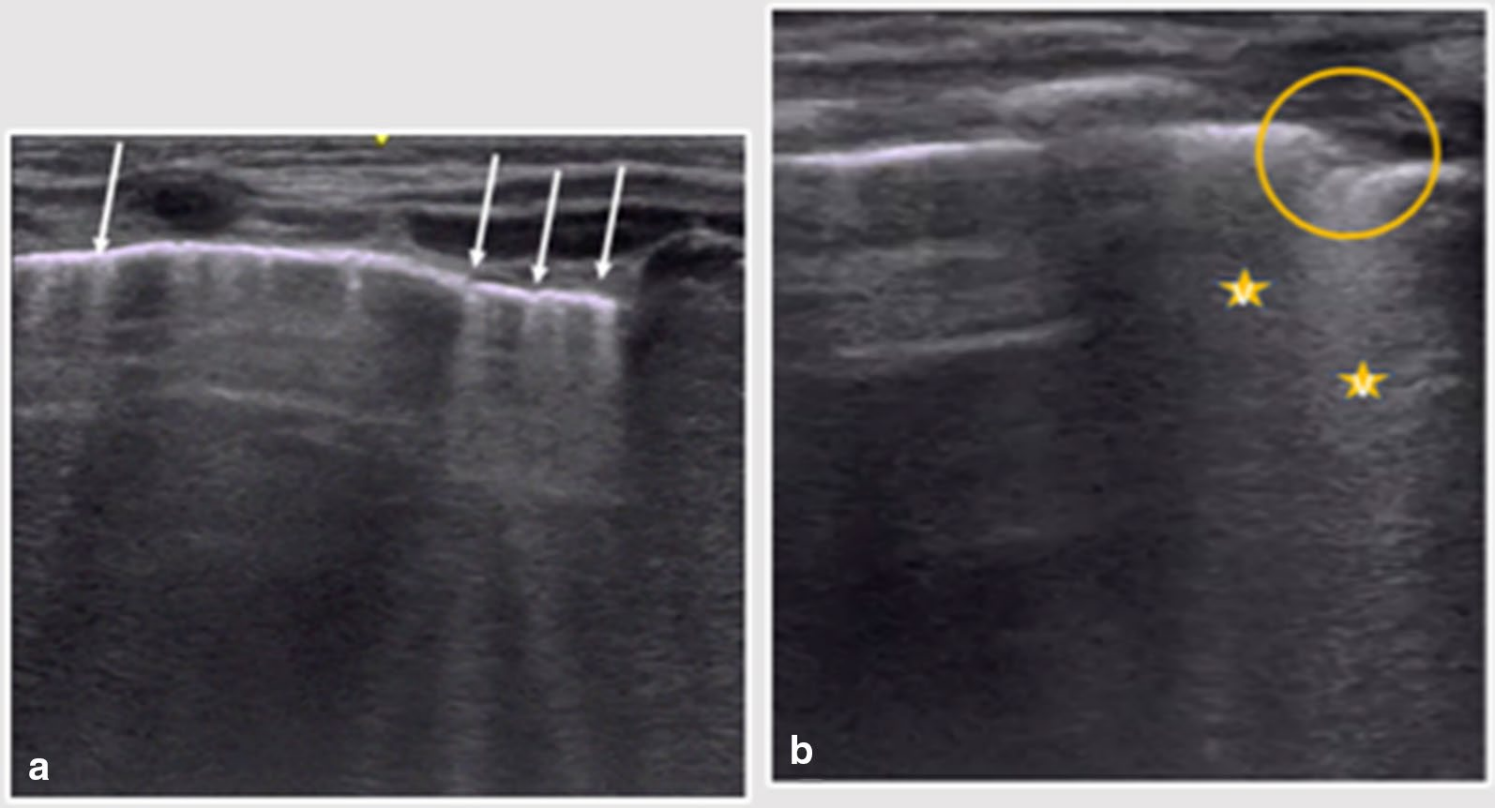
Two-week-old male full-term newborn with fever and mild respiratory symptoms. Lung US showed multiple vertical B-lines on all the lung fields bilaterally (arrows in **a**). Coalescent B-lines could be seen on the posterior and inferior lung fields (stars in **b**), as well as an irregularity of the pleural line and a tiny subpleural consolidation (circle in **b**)

**Fig. 3:**
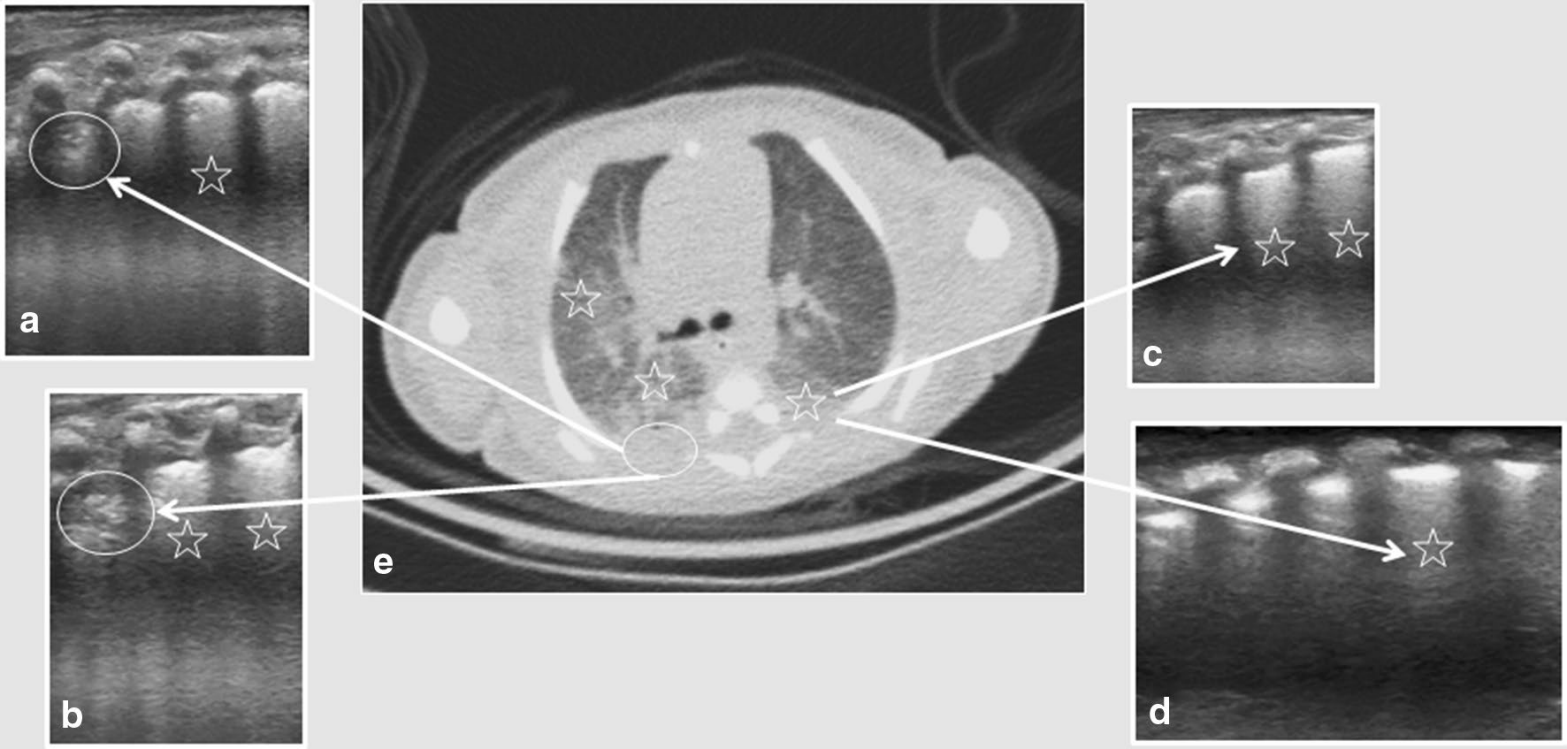
Thirteen-day-old male preterm newborn, presented with hypoxemia, tachypnea and high D-dimer level. Lung US (**a**–**d**) showed irregularities of the pleural line and small subpleural consolidations in the posterior right lung field (circles in **a**, **b**). Confluent B-lines (stars in **a**–**d**) were also present in the bilateral posterior and inferior lung fields corresponding to the chest CT (**e**) ground-glass pattern (stars in **e**). Chest CT showed good correlation with lung ultrasound findings (arrows in **a**–**e**)

Figures 1, 2 and 3 and Additional file 1: Figure S1) show images from four cases in which lung US had provided information on lung injury due to COVID-19.

## Discussion

Since the first reports on COVID-19 pneumonia, emphasis has been given to the role and impact of thoracic imaging for accurate assessment of lung compromise and timely detection of complications. Chest CT has gained an important role in this setting, because chest radiograph is of limited value for COVID-19 diagnosis, with a reported sensitivity of only 69% [95% CI 56–80%] [20]. Lung US has an already established accuracy and reliability in diagnosing many lung pathologies, but up to now, only few studies have been published on its applicability in COVID-19. For pneumonia and ARDS, it is an excellent method of diagnosis and monitoring and has been found to detect lesions not seen on the chest radiograph, especially those localized in the retro-cardiac or juxta-diaphragmatic region [21–23].

Preliminary lung US studies on adult patients with COVID-19 identified numerous B-lines and subpleural pulmonary consolidations in an asymmetric multilobar distribution, involving mainly the lower lobes. What seems to be characteristic of COVID-19 in a positive epidemiological context is the bilateral patchy distribution of multiform clusters alternating with spared areas. Those findings were highly consistent with the findings on CT [24–29]. The first few case reports and case series published in children with COVID-19 showed lung US findings similar to what was described in adults [30–32].

Our findings are in accordance with previous small pediatric reports and adult studies and we also found an apparently good consistency between lung US and CT in terms of findings and topography. Confluent B-lines were later seen as consolidations on a chest CT performed the following day only in two specific zones. We cannot say whether it was a misdiagnosis or an early sonographic identification of organizing pneumonia. This could be clarified only by a study aiming to compare lung US and CT when both are performed simultaneously. Even though some comparisons were made between CT and lung US, this study was not designed to pair up these imaging modalities due to ethical concerns about unnecessarily exposing children to ionizing radiation.

As far as we know, our study was the first in pediatric COVID-19 population to analyze lung US aeration scores. Most of our patients that classified as moderate and severe/critical, had major abnormalities on lung US, and consequently higher lung US aeration scores. Due to the small sample size, we do not have statistical power to confirm the lung US aeration score as a disease severity predictor, but this preliminary result is an important finding that suggests that this score might be an additional tool to help clinicians in risk stratification and resource allocation. Two patients with moderate disease and normal US had an obstructive airway disease reversed with corticosteroids and bronchodilators.

Despite the apparent good association with disease severity, five of our children had significant lung US abnormalities, elevated scores, but few or no respiratory symptoms. All of them were under 6 months of age and four also had elevated D-dimers, which is believed to be a severity marker of disease in adult patients [33]. One of them, a 2 week-old newborn with fever, also had a chest CT that confirmed lung involvement. The reason why these young infants with confirmed lung involvement on imaging and elevated D-dimer have such a mild disease is yet to be clarified as many other clinical aspects of COVID-19 in children.

There are several reasons why we believe lung US may be a promising tool in COVID-19, especially in the pediatric population. First, although other viruses such as the respiratory syncytial virus and parainfluenza virus cause pneumonia lesions that are mostly distributed along the bronchial tree, studies addressing CT findings in children with COVID-19 showed that the periphery of the lung in the subpleural region is the most commonly affected area [34, 35]. Of 43 patients with CT abnormalities due to COVID-19 reported by Ma et al. [3], 95% had a predominance of lesions in the subpleural area and in the lower lung lobes (65%), especially in the posterior segment (78%). Given that the subpleural area seems to be the target for COVID-19, lung US may assume a key role in the early detection of these lung involvements as it easily identifies infections extending to the visceral pleura [16]. Second, chest CT should be followed by complex decontamination procedures and requires transporting sometimes critically ill patients to the radiology suite, while lung US can be performed at the bedside and given its smaller size, would be easier to decontaminate [35]. Of note, two of our critically ill patients were too unstable to leave the PICU and initial lung imaging assessments were made by lung US. Third, while US is radiation-free, CT scan exposes pediatric patients to harmful ionizing radiation during a time at which they are believed to be most at risk of harm [11].

Limitations of this imaging modality still exist, including an inability to visualize centrally located consolidation, inability to differentiate consolidation from atelectasis, and possibly some degree of overdiagnosis, as US can detect even small consolidations of unlikely significance [36–38]. Moreover, the aforementioned assumption from adult studies that lung US in COVID-19 may have characteristic features has not yet been extrapolated to children, as they more frequently have lower respiratory tract disease caused by a variety of viruses that may have a similar pattern. Further studies are needed to better understand this.

Our study also has some limitations. It is a descriptive study with a small number of patients included; however, given the scarcity of available data on lung US findings in COVID-19 pediatric patients, the information provided by our study is relevant and may provide a better understanding of this topic. Sonographers were not blinded to clinical information, because lung US assessment is performed on a regular basis as an extension of the physical examination in our institution. Also, even though the lung US score applied in our patients have been used in many lung pathologies, it is not specific to COVID-19 lung disease as the one proposed by Soldati et al. [39]. Lastly, we could not reliably compare lung US with the gold standard chest CT, since not all patients had both exams or had them performed at the same time. Despite these limitations, as far as we know our study on point-of-care lung US findings in children with COVID-19 is the first to include a more extensive number of pediatric patients. We believe our report adds important information regarding the utility of lung US in pediatric population with COVID-19.

## Conclusion

Point-of-care lung US is a reliable tool that is capable of accurately diagnosing and monitoring many pulmonary conditions. Our study shows that it might also have a key role in children affected by COVID-19, providing early and reliable identification of pulmonary involvement. It performs better than chest radiograph and seems to have a good correlation with chest CT, although further studies are needed to confirm this. Integrated with clinical evaluation, lung US allows a better and rapid characterization of the disease, without ionizing radiation exposure.

## Supplementary information

**Additional file 1: Figure S1.** Female, 11-year-old child with multisystem inflammatory syndrome in children (MIS-C) related to COVID-19 who developed cardiac failure and died. Post-mortem analysis detected SARS-CoV-2 RNA in cardiac and pulmonary tissues by RT-PCR and microthrombotic disease on lungs. Lung ultrasound (LUS—A and C) and Chest computed tomography (CT—B) were performed. There was pleural effusion (PE in A and B) and subpleural consolidation (star in A and B). Confluent B-lines (CBL in C) were also present in LUS in correspondence to the chest CT ground-glass pattern (GGP in B).

## Data Availability

The datasets used and/or analysed during the current study are available from the corresponding author on reasonable request.

## Abbreviations

COVID-19: Coronavirus disease 2019
US: Ultrasound
CT: Computed tomography
PICU: Pediatric Intensive Care Unit
RT-PCR: Reverse-transcriptase-polymerase-chain-reaction
ARDS: Acute Respiratory Distress Syndrome
PED: Pediatric Emergency Department
PEM: Pediatric Emergency
