# Supplementary figures and images for: Point-of-care lung ultrasound imaging in pediatric COVID-19
Source: Ultrasound J. 2020 Nov 30;12:50. doi: 10.1186/s13089-020-00198-z (PMC7702205; doi:10.1186/s13089-020-00198-z)

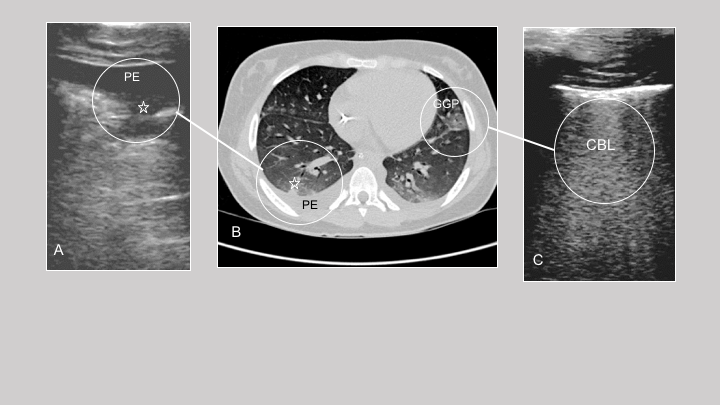

Supplement: Supplementary file 1 — Additional file 1: Figure S1. Female, 11-year-old child with multisystem inflammatory syndrome in children (MIS-C) related to COVID-19 who developed cardiac failure and died. Post-mortem analysis detected SARS-CoV-2 RNA in cardiac and pulmonary tissues by RT-PCR and microthrombotic disease on lungs. Lung ultrasound (LUS—A and C) and Chest computed tomography (CT—B) were performed. There was pleural effusion (PE in A and B) and subpleural consolidation (star in A and B). Confluent B-lines (CBL in C) were also present in LUS in correspondence to the chest CT ground-glass pattern (GGP in B). [file 13089_2020_198_MOESM1_ESM.tiff]
